# Breast screening interval and the characteristics of screen-detected cancers

**DOI:** 10.1186/bcr3758

**Published:** 2015-11-05

**Authors:** Carl A Tupper, Anthony J Maxwell, Susan Astley, Megan Bydder, Soujanya Gadde, Elaine Harkness, Yit Y Lim, Mary Wilson, Julie Morris

**Affiliations:** 1 University of Manchester Medical School, Manchester, UK; 2 Nightingale Centre and Genesis Prevention Centre, University Hospital of South Manchester, UK; 3 Centre for Imaging Sciences, Institute of Population Health, University of Manchester, UK; 4 Medical Statistics Department, Education and Research Centre, University Hospital of South Manchester, UK; 5 Centre for Biostatistics, Institute of Population Health, University of Manchester, UK

## Introduction

There is little evidence regarding the optimum interval between mammograms in a population breast cancer screening programme. The UK NHS Breast Screening Programme employs a 3-year interval, unlike other countries which screen more frequently. This study uses variations in the screening interval within a single large UK screening service to examine possible relationships between screening interval and screen-detected cancer characteristics.

## Methods

A total of 1107 women diagnosed with breast cancer on incident screening over a 5-year period were included. Age, time since last mammogram and surgical histopathology data (tumour type, size, grade, nodal stage, receptor status) were recorded. The Nottingham prognostic index (NPI) was calculated for invasive cancers. Analysis with Spearman's rho and Pearson's correlation was performed.

## Results

The median patient age was 63. Most screening intervals were between 800 and 1200 days. The proportion of women with ductal carcinoma in situ (DCIS) decreased significantly with increasing screening interval, from 24/94 (25.5%) for < 2.5 years to 35/240 (14.6%) for ≥3 years (*p *= 0.032). No significant associations were found between other tumour variables and screening interval. The average NPI score was 3.49 with a Pearson correlation coefficient of −0.032 (*p *= 0.389).

## Conclusion

The findings suggest that there is a significant rate of progression of DCIS to invasive disease within the current 3-year screening interval. This, together with the rate and characteristics of interval cancers (which were not examined in this study), appears to be more important in determining the optimum screening interval than the characteristics of the invasive cancers detected by screening.

